# Barriers and facilitators of access to HIV prevention, care, and treatment services among people living with HIV in Kerman, Iran: a qualitative study

**DOI:** 10.1186/s12913-022-08483-4

**Published:** 2022-08-29

**Authors:** Zahra Jaafari, Willi McFarland, Sana Eybpoosh, Seyed Vahid Ahmadi Tabatabaei, Mehdi Shafiei Bafti, Ebrahim Ranjbar, Hamid Sharifi

**Affiliations:** 1 HIV/STI Surveillance Research Center, and WHO Collaborating Center for HIV Surveillance, Institute for Futures Studies in Health, Kerman University of Medical Sciences, Kerman, Iran; 2 Department of Epidemiology and Biostatistics, University of California San Francisco, San Francisco, CA USA; 3 Department of Epidemiology and Biostatistics, Research Centre for Emerging and Reemerging Infectious Diseases, Pasteur Institute of Iran, Tehran, Iran; 4 Social Determinants of Health Research Center, Institute for Futures Studies in Health, Kerman University of Medical Sciences, Kerman, Iran; 5 Deputy for Health, Kerman University of Medical Sciences, Kerman, Iran; 6 Behavioral Disease Counseling Center, Kerman University of Medical Sciences, Kerman, Iran

**Keywords:** Health Services Accessibility, HIV, Qualitative Research, Iran

## Abstract

**Background:**

Low access to HIV prevention, care, and treatment services among people living with HIV (PLWH) is a barrier to the control of the epidemic worldwide. The present study aimed to assess the barriers and facilitators to HIV services among PLWH in Kerman, Iran.

**Methods:**

In this qualitative study, a convenience sample of 25 PLWH who had received HIV prevention, treatment, or care services, and six PLWH who had not yet received services were recruited between August-October 2020. Data were collected using a semi-structured, face-to-face interview. Data were examined by inductive content analysis using MAXQDA 10 software.

**Results:**

Nine categories of facilitators and 11 categories of barriers to HIV services were identified. Facilitating factors included: maintaining health status, feeling scared, trust in the health system, how they were treated by service providers, provision of suitable hours by the service provider center, changing attitudes towards HIV in society, acceptance of the disease by the patient's family, hope for the future and feeling the need for consulting services. Barriers included financial problems, side effects and belief in efficacy, distance and transportation problems, fear of being recognized, stigma towards PLWH, organization of services, improper treatment by service providers, unsuitable hours by the service provider center, lack of trust in the health system, lack of family support, and inadequate or low-quality service.

**Conclusion:**

Many facilitators and barriers to HIV prevention, treatment, and care are amenable to change and better management by healthcare and service providers. Addressing these factors is likely to increase the willingness to use services by those who have never previously accessed them.

**Supplementary Information:**

The online version contains supplementary material available at 10.1186/s12913-022-08483-4.

## Background

Access to HIV care may be the most important step in reducing morbidity and mortality of people living with HIV (PLWH) and preventing onward transmission [1, 2]. PLWH on antiretroviral therapy (ART) who achieve low levels of the virus can have a full life expectancy [3]. PLWH with low viral loads also have a low risk of transmitting the virus to others [2]. In addition, access to voluntary counseling and testing (VCT) for HIV can avert much morbidity and mortality by avoiding late diagnosis of patients with severe immune system damage and shortening the time of possible transmission to others [4]. Therefore, ensuring easy access to VCT services for persons at risk for HIV is also needed to end the epidemic [5]. Research has identified many challenges that disrupt HIV care at multiple levels, including at the individual, societal, and health systems levels. For example, factors range from denial of infection or HIV’s existence, refusal to get tested, refusal to take ART, fear of breach of confidentiality, stigma, discrimination, racism, and poverty, to stock-outs of HIV tests and viral load testing reagents [6–8].

To gauge progress in maximizing participation in HIV care, a framework of several stages, known as the HIV treatment cascade, has been developed. The HIV treatment cascade includes testing and diagnosing HIV, linkage to treatment, initiating and adhering to ART, and sustaining viral suppression [9–11]. Projections indicate that HIV will be controlled if at least 90% of infected people are aware of their disease, 90% of those who are aware of their HIV status receive sustained ART, and 90% of those on ART remain virally suppressed [12]. To end the epidemic, UNAIDS has announced 95–95-95 targets for 2030; that is, elimination of HIV transmission can be achieved if at least 95% of PLWH are diagnosed, at least 95% of those diagnosed are on ART, and at least 95% of those on ART are virologically suppressed [13]. As of the end of 2020, the World Health Organization (WHO) estimated there were 37.7 million PLWH worldwide [14]. About 84% of PLWH were aware of their disease status, 73% received ART, and 66% achieved HIV suppression. However, these gaps in HIV care still resulted in 680,000 people dying of AIDS and 1.5 million people infected with HIV in 2020 [14].

Progress on the HIV response in the Middle East and North Africa (MENA) region lags behind the rest of the world. The HIV epidemic is, in fact, on the rise in the MENA. It is estimated that new HIV infections in 2019 increased by 25% over new infections in 2010. The HIV treatment cascade is far from achieving levels needed to control the HIV epidemic, with only 38% of PLWH accessing treatment in 2019 and 8,000 dying from AIDS-related causes [15]. In Iran as of 2019, there were an estimated 59,314 PLWH, of whom 22,054 were diagnosed and alive. By the end of 2019, 15,949 PLWH had used services, of who 14,685 were taking ART. Therefore, the HIV treatment cascade in Iran has severe gaps in the diagnosis and treatment stages [16].

To improve access to HIV prevention, care, and treatment services, it is necessary to know the strategies and interventions that can successfully link PLWH to services. These barriers and facilitators could be specific for each region, country, and population. Moreover, PLWH themselves may have the best insight on the specific facilitators and barriers to HIV services through their lived experiences. Therefore, the present study was designed to assess the barriers and facilitators of HIV prevention, care, and treatment services in Kerman, the largest city in southeast Iran, through interviews with PLWH who have and who have not accessed these services.

## Methods

### Study population and setting

This qualitative study included key informant interviews from two groups of PLWH. The first (group A, *n* = 25) was PLWH who had been receiving HIV prevention, care, and treatment services from a VCT center which also provides HIV care and treatment. The second (group B, *n* = 6) were PLWH who had not previously received HIV prevention, care, and treatment services recruited by a non-governmental organization (NGO). These persons include those referred to NGOs to receive non-HIV-related charitable assistance, such as necessities of life (e.g., food packages) and hygiene and health items (e.g., detergents and disinfectants). NGO staff introduced the study, stating the objectives of the research, and if willing were referred to the researchers for inclusion in the study. Recruitment occurred between August and October 2020. Inclusion criteria were adult PLWH who had and or had not received services and consented to participate in this study. Recruitment continued during analysis until information saturation was surpassed such that no new information was found (see below).

The collaborating VCT center provides services such as counseling and testing for clients at risk for HIV and care and treatment of persons living with HIV. The VCT site takes multiple measures to protect the privacy and prevent discrimination against PLWH. The main clients of this center are people with high-risk behaviors (e.g., people who inject drugs, female sex workers, men who have sex with men), couples testing for HIV who intend to get married, and pregnant women, including those with HIV [17]. Services provided to PLWH include identifying the basic needs of clients, helping them access needed services, navigating and maintaining communication with clients, and ensuring medical other services such as ART under the supervision of infectious disease specialists, psychiatric care, HIV/STI prevention, methadone maintenance, and sterile syringes and supplies to people who inject drugs [18].

The collaborating NGO is located in Kerman city and serves the general public, people at high risk for HIV (for example, people who use drugs and sex workers), and PLWH. Among the activities of this organization are life skills and resilience training, a free and confidential hotline line, HIV educational courses for governmental and non-governmental institutions, rapid and confidential HIV testing at the NGO and at high-risk locations (e.g., parks and a gathering place for persons at risk), empowerment and employment training for women living with HIV, and provision of medical supplements and food baskets with the aim of strengthening the immune system.

### Procedures

This qualitative study was conducted by a research team specializing in HIV who were not involved with providing services to the study population. Before the interview, the female trained interviewer (i.e., the first author) and a female graduate student assistant went to the interview sites to learn more about the facilities and the population to be interviewed. Potential participants were referred by VCT staff, screened for eligibility and interest, and provide with information to obtain informed consent by the interviewer team.

A stipend of 500,000 Iranian reals (~ $3 USD) was given to the participants for travel expenses and their time to participate in the study. The interviews were conducted face-to-face, in Persian, using a semi-structured interview guide in a quiet room with only the interviewee and the researcher present. The duration of each interview was between 30 and 35 min.

The interview guide comprised of open-ended questions according to the objectives of the study. Many of the questions were obtained from the literature review [19, 20], others were obtained from local experts in the field of HIV, and some were added during the interview. All questions, including the initial and those subsequently added, were reviewed by three experts in the field. Examples of questions include for group A: “What are the reasons for you to go to the VCT center to receive services?”, “What do you think are the barriers to receiving services from VCT Center?”, “Do you have full access to the VCT center in terms of distance and means of transportation?”); and for group B: “What are the reasons why you did not go to the VCT center to receive services?”, “Are you worried about meeting people you know when you go to the VCT center and let them know about your illness?” In addition to the audio recording, additional notes were written by the interviewer.

### Data analysis

Data were analyzed using the inductive content analysis approach [21]. Recorded interviews were transcribed verbatim on the day of the interview by the interviewer. Data were analyzed and interpreted at the time of transcription and iteratively through multiple readings by three authors (ZJ, SE, HSH). Consensus on the key themes was sought through group discussions with all authors, resolving any cases of ambiguity delineating themes. The texts were transferred to MAXQDA 10 software for data management and analysis. The data were systematically coded in the form of text with patterns and themes identified by one of the researchers (first author). The coding process in the software was such that the interview files were first entered into the software and pasted in separate folders. The researcher then started coding after reading the text of each folder. The codes were grouped with common concepts. One of the capabilities of this software is having a positive sign next to the categories with subcategories used for coding in this analysis. During coding, the researcher shifted the order of the codes and placed the codes with common concepts in a category with a specific color. Also, while moving the codes with common concepts, the researcher paid attention to the frequency of each code that is displayed in the software with numbers next to the codes. The research team held weekly meetings to verify the codes and subcategories, and any ambiguity was resolved. Sampling continued until information saturation was achieved, and no new concepts were identified during the analysis. In addition, to be confident about information saturation, three more respondents were interviewed in both groups after information saturation was deemed achieved by the investigators.

### Trustworthiness

In this study, we used four criteria (credibility, transferability, dependability, and conformability) to ensure trustworthiness [22]. The study site was visited before the interviews and data collection to address the credibility criterion. Data credibility was also established by reviewing the adequacy of the interviews and confirming interpretations obtained from the interviews. To examine the transfer criterion, an attempt was made to describe the characteristics of the participants in detail and consider using maximum variability in sampling. To address the dependability criterion, study processes were described to the team in detail, and interviews were audited externally; in this sense, the opinions of foreign observers were used. Discussions in research team meetings raised additional issues that were considered for confirmability.

## Results

Among the 25 respondents recruited from the VCT center who had used HIV services (group A), the majority were male (60%), 41–50 years old (64%), divorced or widowed (52%), high school or less (72%), and had a monthly income of less than 20 USD (72%), and (Table 1). Six respondents were recruited for group B (i.e., had not previously received HIV services). All six were male and had education in high school or less (100%), and most were 41–50 years (83.3%) and single (83.3%), half had a monthly income of less than 20 USD and the other half had between 20 to 40 USD. In group A, most participants were classified in other occupations (except unemployed and housewives) (40%). In the occupation classification of group B, most of the participants were unemployed (66.6%).

**Table 1:** Demographic characteristics of key informants on barriers and facilitators of HIV prevention, care, and treatment services, Kerman, Iran, 2020 (*n* = 31)

Variable		Recipients of services n (%)	Non-recipients of services n (%)
Age	30–40	2 (8.0)	1 (16.7)
	41–50	16 (64.0)	5 (83.3)
	51–60	7 (28.0)	-
Sex	Male	15 (60.0)	6 (100.0)
	Female	10 (40.0)	-
Marital status	Married	7 (28.0)	-
	Single	5 (20.0)	5 (83.3)
	Separated/Widowed	13 (52.0)	1 (16.7)
Education	High school or less	18 (72.0)	6 (100.0)
	High school diploma or greater	7 (28.0)	-
Occupation	Unemployed	6 (24.0)	4 (66.6)
	Housewife	9 (36.0)	-
	Other occupations	10 (40.0)	2 (33.3)
Monthly income	< $20	18 (72.0)	3 (50.0)
	$20–40	6 (24.0)	3 (50.0)
	> $40	1 (4.0)	-

Through analysis of the respondent interviews, we identified nine themes for facilitators of HIV prevention, care, and treatment services and 11 themes as barriers. The following summarizes these themes. Quotations are ascribed to respondents using pseudonyms. The most important themes of the facilitators based on the repetitions in the interviews were maintaining health status (20), feeling scared (18), trust in the health system (15), how they were treated by service providers (10), provision of suitable hours by the service provider center (5), changing attitudes towards HIV in society (3), acceptance of the disease by the patient's family (3), hope for the future (2), and feeling the need for consulting services (2). However, the most important themes of the barriers were: financial problems (25), side effects and belief in efficacy (19), distance and transportation problems (17), fear of being recognized (16), stigma towards PLWH (15), organization of services (4), improper treatment by service providers (4), unsuitable hours by the service provider center (3), lack of trust in the health system (3), lack of family support (2), and inadequate or low-quality service (2). In this section, we presented the five themes with the most repetitions and the remaining themes were presented in the Additional fil 1 appendix.

### Facilitators of HIV prevention, care, and treatment service

#### Maintaining health status

Some patients also wanted to continue the treatment process due to the good results they received from following their treatment with ART. Said a housewife: *“I have had this disease for 16 years. All the time, I tried to follow my treatment regularly. My tests show that the virus has been controlled in my body, but I still have to continue my treatment; so that our disease does not recur. I am very happy that I got a good result from my treatment.”* (Shirin, 58, widow, middle school).

#### Feeling scared

Despite regular visits to the VCT center, some respondents remained concerned about the recurrence of their disease, which motivated regular follow-up visits to receive their medication. A man whose wife had died said, *“I feel better when I take my medication. Certainly, if I stop taking my medication and do not continue my treatment, I will face problems, and my health will be endangered, and my illness will recur.”* (Hossein, 45 years old, widower, primary school education level).

When asked about the reason for referring to VCT and following up on treatment, some respondents expressed fear of death with a visible somber expression. Said one male respondent, *“I take my medicine because I am afraid of death. If I do not take my medication and follow my care, I will lose myself, there are many PLWH who do not take medication and eventually die. If I do not take my medicine, I will eventually die.”* (Sadegh, 38 years old, single, high school diploma).

Some respondents felt responsible for the health of others, and this increased their desire to pursue their treatment. Said one man, *“Taking medication helps us stay healthy. Because I am taking my medicine, I am in very good health. Taking medication prevents HIV from being passed on to others. I'm always worried that I might make someone else sick, and that’s why I always take my medicine.”* (Hadi, 57 years old, divorced, high school diploma).

Some participants were also worried about their spouses becoming infected. Said a married woman, *“When I wanted to get married, my husband and I went to VCT, and the counselor explained to my husband about the ways the disease was transmitted, and now that we are married, although my husband uses a condom during sex and does not have HIV; my husband always goes to VCT to get checked and make sure he is negative.”* (Nastaran, 49 years old, married, high school diploma).

Some female respondents expressed the responsibility of motherhood and were terrified of the displacement of their child following their own death; in the event, none of the men expressed this. A woman who was a divorced housewife stated that the only reason she went to VCT to get the medicine was the existence of her child, *“When I heard the news of my illness, I was very shocked and said to myself, why me? why me? … and I just wanted to die. I did not want to continue living. I was so bad that it could not be described. I was called by the VCT center and told to come to the center for treatment, but I had made my own decision; I just wanted to die. The staff of the center said that if you do not take your medicine, you will die. I thought to myself, what will happen to my boy if I die? Will he continue my path? … I was very worried about his future, and I was afraid that he would be displaced after me. After all, I am a mother. At the moment, my only motivation is to continue my treatment and keep my child alive. I want to survive because I am afraid of my child's future and I want him to take the right path; I hope I can do these responsibilities well.”* (Mahsa, 42 years old, divorced, middle school education).

#### Trust in the health system

An important factor for respondents recruited from the VCT center was to keep their information private and to not disclose it elsewhere. One female housewife respondent said, “*Given that one of my relatives works at the VCT center, I was very concerned that others would find out the details of my illness through my relatives. But the staff of the center talked to me and said that there is no problem and no one will disclose your information, and if someone does that, he will be fired. So far, there has been no problem in this regard, and no information has been revealed from us anywhere else.”* (Sima, 47 years old, married, primary school education).

#### How they were treated by service providers

Respondents mentioned how they were treated by service providers as a positive reason to continue treatment. One female respondent said, “*The behavior of the VCT staff is really great; when I go there, they treat me warmly and friendly. It has happened several times that they call me a few days before I finish my medication and remind me to go to the center and receive the medication.”* (Elmira, 30 years old, married, middle school education).

#### Provision of suitable hours by the service provider center

Many respondents’ financial situation meant that they needed to walk to the VCT center for services. Therefore, the center's working hours were an important factor in their access to services. Said an unemployed male respondent, *“Currently, the center is open in the morning, and I think this is a better time to provide services to patients. Because many patients come to the center on foot, and if the working hours are in the afternoon, the heat bothers us in the summer, and in the winter, it gets dark very quickly, and we who want to go to the center on foot face problems.”* (Amir, 56 years old, widower, high school diploma).

### Barriers to access to HIV prevention, care, and treatment services

#### Financial problems

When respondents were asked about barriers to accessing services, many cited financial problems such as lack of travel fare. Said one male respondent, *“My place of residence is in the welfare dormitory. I do not have a job that I can earn. The fare for the car is high. When I go to the center, I have to walk, which really bores me.”* (Sadegh, 38 years old, single, high school diploma).

According to some respondents, drug addiction can aggravate the economic barrier. A male respondent said, *“Among people with HIV, I know people who are also addicted to drugs. These people usually do not have a job to earn money, and even if they have a small income, they spend it on drugs, and there is no money left to go to the center and receive services.”* (Ehsan, widower, 58 years old, illiterate).

#### Side effects and doubts on efficacy

People in group A took their medications and care of themselves for fear of viral rebound and death, and were satisfied with the outcome of their treatment. People in group B were less likely to seek treatment for diverse reasons, including drug side effects. Many respondents who had not previously accessed services spoke about their experience with taking medication and their side effects. Said one unemployed male respondent, *“I used to go to the center to get medicine. But when I took the medication, I felt dizzy. I was drowsy and wanted to sleep. I could not do anything at all. For this reason, I did not continue taking the medication. The doctor told me to continue taking medicine until your body got used to it, but I could not bear the side effects of medicine and I did not continue taking the medicine.”* (Parsa, 41 years old, single, middle school education).

Some also spoke of a lack of confidence in the effectiveness of drugs. A housewife respondent said, “*I ask you (the interviewer), do these drugs affect the recovery of patients? … I have seen many people take drugs related to HIV and eventually die. So, what is the use of taking medicine like a moving doll and eventually dying?”* (Mahsa, 42 years old, divorced, middle school education).

#### Distance and transportation problems

Some respondents cited distance and transportation problems as a barrier to accessing services. A female respondent said, *“There is only one VCT center in our city. The location of this center is far from the residence of many patients. Therefore, the means of transportation are too few to go to the center because it is far from the residence of some patients. So if there were several VCT centers in our city, it would definitely be easier to get around.”* (Sahar, 42 years old, married, high school diploma).

#### Fear of being recognized

Many respondents expressed concern about being identified by different people. One housewife respondent said, “*I do not want anyone to know about my illness from my relatives because they do not have a good attitude towards this disease. Since one of my relatives has HIV, he goes to the VCT center. But I do not want him to know that I also go to this center. For this reason, when I want to go to the center, I call first; if someone who is a relative of mine has an appointment to visit that day, I will not go to the VCT center that day.”* (Nastaran, 49 years old, married, high school diploma).

Some respondents also called for separating the VCT center for PLWH from the general population. Said one male respondent, *“When we go to the center to receive services, there are other people in the center, for example, students who have come for education or couples who are planning to get married and come for testing. When we pick up our medical records and go to the doctor's room, the other people in the center become aware that we are PLWH and look at us in a special way, thus identifying us among different people. But if there were two separate centers for PLWH and the general population, there would be no such problems.”* (Arash, 48 years old, married, Bachelor’s degree).

#### Stigma towards PLWH

Many respondents complained of HIV-related stigma in the community. Said one housewife respondent, *“I worked in the hospital reception. We separated a few years ago because of my husband’s addiction. My mother-in-law did not want to take care of her son (my ex-wife) and therefore wanted to force us to remarry. She came to my place of work and informed the director of the hospital about my illness. I thought to myself that they might test me, so I had to be honest and tell the truth to the hospital director. I told the truth، but I said I followed the health tips, my boss objected to me staying at work and said that if a needle gets in your hand while pinning the medical records of patients, it is dangerous and other staff may be infected and therefore I lost my job.”* (Nastaran, 49 years old, married, high school diploma).

Some also objected to the name of the VCT center as stigmatizing. A female respondent said, “*When I take a taxi to the VCT center to receive services, I tell myself that the entrance sign of this center says "Behavioral Diseases Center." Maybe the taxi driver who took me see me entering the center, say to others, what is wrong with this person who came to the center? … I wish the name of this center was something else.”* (Nastaran, 49 years old, married, high school diploma).

## Discussion

In this study, we interviewed two groups of PLWH about the facilitators and barriers of prevention, care, and treatment services. Results showed that patients are motivated to seek HIV treatment and related services by fear of HIV and its consequences, hope for the future, maintaining health status, and the need to address other concerns in the personal lives of PLWH. However, major barriers to accessing services included practical concerns, such as financial problems, disorganization of diverse services, distance and transportation, the inadequacy of the laboratory and supplies of HIV-related and non-related medications, and hours of operation. Other fears were related to stigma, such as fear of being recognized in the community, and protection of their privacy. In addition, some issues were both facilitators for some and barriers for others, such as positive or negative attitudes of staff, having or lacking family support, hours of operation, and trust or lack of trust in the health system particularly with respect to privacy. It was encouraging that many respondents felt educating the public about HIV caused an improvement in stigma over time. Notably, concerns about side effects and the efficacy of HIV treatments persisted despite decades of improvements in antiretroviral medications.

Fear was often mentioned during the interviews with PLWH, including fear of death, fear of viral rebound, fear of transmitting the disease to others, and fear of displacing their children in the absence of parents. Our findings are echoed elsewhere in the world. A study in Mississippi found that perceived vulnerability to negative consequences (e.g., progression of HIV to AIDS and death) has been a factor in PLWH seeking a referral for treatment [23]. Considering that ART prevents opportunistic infections and also reduces the proliferation of HIV [24], treatment should help address the fear of the consequences to the patient and fear of transmitting to others. ART can improve the health and wellbeing of PLWH and thus enable them to make a living. Providing HIV treatment together with programs to improve quality of life should therefore encourage PLWH to initiate and be retained in care. Our data point to the need to address the social welfare, the physical and mental health of PLWH [25], including improving family reactions which can be a source of stress or support if properly managed [26]. As in the present study, some participants discussed HIV care as providing assistance to deal with problems in their personal lives. A previously qualitative study in Iran showed that if there are counseling services available to PLWH, including group counseling sessions, they can more readily join the society [27].

We found that financial problems are a major barrier to accessing HIV services, with many respondent complaints. Other studies also indicated that financial problems are barriers to adherence to ART. For example, a study conducted in the United States found travel expenses to be a barrier to follow-through on referrals to service centers and to filling prescriptions [28]. Financial problems of PLWH can also affect other people. For example, a study in Iran showed that women with high-risk sexual behaviors resulting from financial need and lack of support receive more money if they do not use condoms during sex [29]. Therefore, addressing the financial problems of PLWH can provide wider-reaching benefits to themselves and their partners.

A pillar of HIV prevention is that PLWH need to be aware of their infection to receive care and reduce onward transmission [14]. Therefore, it is important to identify the factors that prevent the linkage of PLWH to HIV counseling and testing, to initiate care and treatment, and to be retained on their treatment plan [30]. Notable barriers found in the present study were to structural factors, including the hours of operation, distance to services, having enough service sites, and colocation of diverse services. A review of studies corroborates such barriers in a wide range of middle-income countries, particularly among adolescents living with HIV where linkage and retention has been low [31]. Other studies have shown that regular leave requests from employers to visit service centers have led to job losses [32]. Due to the fact that the working hours of government offices in Iran are from 8 AM to 2 PM, having hours of operation only in this time range is an obstacle for many patients who are unable to leave their place of work. Changing the working hours of the service provider center or providing services earlier in the morning and later in the evening may provide better conditions for patients who want to use the service. Considering HIV as a chronic disease which may occur in conjunction with other chronic diseases, such as asthma, diabetes, etc., extending hours and collocating with other health services would further reduce demands on time.

Immune dysfunction that results from chronic HIV infection can be stopped and reversed by initiating ART. Although ART can successfully suppress viral replication, the side effects of these drugs can be a serious problem that reduces the effectiveness of treatment [33, 34]. Side effects of ART are common, but in most cases, these side effects decrease over time or improve with symptomatic treatment [35]. In this study, many PLWH reported side effects of medications such as nausea and dizziness. In this regard, a study conducted in Uganda of the target population of pregnant women with HIV showed that patients were afraid of the large size of the pills and felt uncomfortable after taking the medications because of the bitter taste of the pills [36]. Simplifying treatment, including the use of alternative drugs, as well as reducing the frequency and dose of medication, can reduce the unpleasant symptoms of the drug and improve adherence to the treatment plan [37]. Also, educating patients and explaining to them about the benefits of ART to improve their health and quality of life may have beneficial effects on keeping PLWH in treatment.

Finally, our study overwhelming points to the need to ensure the privacy of patient information coupled with reducing HIV stigma among staff and society at large. Disclosing and discussing patients’ HIV status and not maintaining the confidentiality of information were primary concerns of PLWH interviewed and barriers to attending HIV services. Studies in India and South Africa showed that many people did not return for further treatment after testing positive and informed of their infection because of the fear of disclosing information about them in their nearby communities [38, 39]. Stigma can be due to insufficient knowledge about the disease and due to norms in how society views the sexual or drug injection modes of transmission. Inadequate knowledge of HIV combined with the fear of getting infected can lead people to withdrawal from PLWH [40]. Further, PLWH will be fearful of presenting for prevention and care services and of sharing their status with their support networks others and health workers [41]. Therefore, putting strict confidentiality measures in place, training staff on procedures, and enforcing consequences for breach of confidentiality will help reassure patients about the privacy of their information. Meanwhile, providing training for health care providers and the public at large can help reduce the stigma associated with HIV [42].

### Limitations

This study has three limitations. First, the study was based on a convenience sampling in only one VCT center in one city. Therefore, generalizability may be limited, and other studies elsewhere in the country may show different results for different regions. Second, people who did not receive services were less accessible to us. We believe our recruitment of six participants who did not receive services provided saturation for our key themes presented here. Nonetheless, we recognize the potential limitation of relying on fewer informants in this critical category, particularly women. Third, we did not collect data on the different, specific sub-populations at risk for HIV (i.e., sex workers, people who use drugs) and the duration of HIV infection. These factors need to be considered for future studies.

## Conclusion

This study highlighted many barriers for PLWH to use the services provided. Barriers to services not only increase patient morbidity and mortality, they also undermine recent advances in testing, treating, and suppressing the virus in patients to prevent onward transmission. Understanding these barriers in greater detail can help improve each step in the treatment cascade of HIV in Iran. Our findings provide information for policymakers to develop effective strategies to combat the spread of HIV, particularly through treatment adherence. We believe that the process of caring for and treating PLWH will be more successful if these barriers are addressed; especially by removing the fear, stigma, and increasing trust to health services through some actions such as education. Also, our study showed that understanding the benefits of treatment is effective in linkage them to treatment; so increasing the knowledge of the PLWH in this regard can be useful. In addition, strategies and service innovative delivery models, for example, remote service delivery, may have a positive effect on overcoming the barriers. Finally, interventions to improve the linkage of PLWH to treatment need a multi-level framework to achieve maximum results.

## Supplementary Information

**Additional file 1.**

## Data Availability

Data will be available upon request submitted to the corresponding author.

## Abbreviations

PLWH: People Living With HIV
VCT: Voluntary Counseling and Testing
ART: Antiretroviral Therapy
WHO: World Health Organization
MENA: Middle East and North Africa
NGO: Non-Governmental Organization
