# Supplementary material for: Barriers and facilitators of access to HIV prevention, care, and treatment services among people living with HIV in Kerman, Iran: a qualitative study
Source: BMC Health Serv Res. 2022 Aug 29;22:1097. doi: 10.1186/s12913-022-08483-4 (PMC9422119; doi:10.1186/s12913-022-08483-4)
Supplement: Supplementary file 1 — Additional file 1. [file 12913_2022_8483_MOESM1_ESM.docx]

**Appendix: Barriers and facilitators of access to HIV prevention, care, and treatment services**

**Facilitators of HIV prevention, care, and treatment services**

**Changing attitudes towards HIV in society.** Some respondents who were receiving services stated that educating the community about HIV has produced a better attitude towards this disease than in the past. A male respondent described his changing experience when hospitalized over the past several years, *“I was hospitalized about ten years ago. Above my bed was written on a board with a red marker: HIV positive. My roommates looked at me with a special look, and some of them were even scared. But recently, when I was hospitalized, there was no more news about the sign and the writing on it, and only a red ribbon was tied to my hand.”* (Sajad, 46 years old, single, high school education).

Some respondents discussed the role of the media in raising public awareness about HIV. A woman who was a housewife said, “*Previously, when someone noticed my illness, they thought that even if they sat on the chair I was sitting in, they would get HIV. But I have not encountered such encounters at the moment. I think because of the propaganda and training that is being broadcast on television, people have become aware and know the ways in which this disease is transmitted.”* (Haleh, 42 years old, married, primary school education).

**Acceptance of the disease by the patient's family.** Some respondents talked about their activities at home and were happy to live with their families despite their illness. Said one male respondent, *“My family is aware of my illness, and they have no problem with it at all. I bathe my niece. I always prepare kebabs for food. I always prepare the salad myself. My family does not really mistreat me.”* (Arash, 48 years old, married, Bachelor’s degree).

**Hope for the future.** Some respondents were optimistic about the future and their treatment. Said one housewife: *“I always think to myself, if I go to the VCT center regularly and take my medication regularly, maybe there will come a time when I will fully recover.”* (Sara, 52 years old, widower, primary school education).

**Feeling the need for consulting services.** Unlike the men in the study, who were reluctant to seek help, women were interested in using counselor guidance. Some respondents had concerns unrelated to HIV services and sought help from a counselor to resolve them. Said a housewife respondent, *“[I] had a dispute with my husband, and when I talked to the center counselor about these disputes, she guided me. My problem is not completely solved yet, but it is better than before.”* (Sareh, 47 years old, married, primary school education).

**Barriers to access to HIV prevention, care, and treatment services**

**Organization of services.** Some respondents noted the organization of services affects access. For example, respondents receiving methadone complained about the distance between the VCT center and the methadone center. A male respondent said, *“There used to be a center that provided both services and methadone to patients, but it is no longer the same as before and they separated the two centers, so there are problems, including we have to go to two centers and the cost goes up, and pay more, if both were done in the same center, we would pay less.”* (Arash, 48 years old, married, Bachelor’s degree).

Some respondents complained that government agencies did not pay attention to HIV. A male respondent said, “*In the community, there are treated better with other patients, such as patients with MS or thalassemia, and financial aid is provided for them, but there is no good view of HIV. Welfare provides financial assistance to other special patients but does not help us at all.”* (Hadi, 57 years old, divorced, high school diploma).

**Improper treatment by service providers.** Although many respondents were satisfied with their treatment by the center staff, a limited number expressed dissatisfaction. A housewife respondent said, *“When I first went to the center, the staff there said that you could call whenever you had a problem, and even if you asked for medicine and you could not come to the VCT center, we would send it your home. I have been feeling fragile for a few days now, and I called the VCT center and expressed my problem. They just said, no problem, and come to the center if you want. But I have serious financial problems, and I have trouble traveling and paying for transportation. In general, the follow-up of the staff is somewhat different from what I expected from them.”* (Mahsa, 42 years old, divorced, middle school education).

**Unsuitable hours by the service provider center.** Although some respondents were satisfied with the working hours of the center, others expressed dissatisfaction. Said one woman, *“I used to work in a restaurant and I was at work all the time that the VCT center was providing services. For this reason, it was very difficult to go to the VCT center and receive services. Many other patients may have similar conditions.”* (Sareh, 47 years old, married, primary school education).

Given that a significant proportion of respondents were housewives, some protested the working hours of the center. Said one female respondent, *“I usually have to do housework in the morning, for example, cooking and tidying the house. That's why I don't have time to leave the house in the morning. The VCT center is open only before noon. It would have been much better if the center had also provided services in the afternoon.”* (Shima, 42 years old, married, primary school education).

**Lack of trust in the health system.** Although many respondents expressed trust in the center staff, others expressed dissatisfaction. A female respondent said, *“Previously, the behavior of the staff was such that if you talked to them about your problems, the next day all the staff knew about your problem. But now, their behavior has improved. However, I do not talk much with the staff and even the consultant because I think if I say something, they will reveal what I say to other staff.”* (Nastaran, 49 years old, married, high school diploma).

**Lack of family support.** Some respondents were deprived of family support after their families became aware of their illness. An unemployed male respondent cried with tears in his eyes when he said, *“When my father found out about my illness. He gave me some money and told me to go and never do not come back home. My father said that you destroyed our family reputation.”* (Majid, 44 years old, single, high school diploma).

**Inadequate or low-quality service.** Some respondents pointed to inadequate or low-quality service. For example, many complained about the lack of drugs other than their HIV-related medications at the VCT Center Pharmacy. Said one male respondent, “*Whenever I needed a medicine other than HIV-related medicine and went to the central pharmacy, the staff said we did not have the medicine you were looking for. If you need it, get it from pharmacies outside of VCT. From out-of-center pharmacies that we cannot afford because we cannot afford to pay for medicines.”* (Behnam, 43 years old, single, primary school education).

Some respondents expressed distrust of the results of the laboratories. Said a housewife respondent, *“I had a test once in another city, and my test showed that my physical condition is optimal. About a month later, I tested in the laboratories offered by the VCT center, but my health was not good. I later found out that the lab tools were faulty and misreported the results.”* (Nastaran, 49 years old, married, high school diploma).
